# 6-Amino-4-(3-iodo­anilino)-2-methyl­pyrimidin-1-ium chloride

**DOI:** 10.1107/S1600536812028401

**Published:** 2012-06-27

**Authors:** Wafaa A. Zaghary, Detlef Geffken, Seik Weng Ng

**Affiliations:** a Department of Pharmaceutical Chemistry, College of Pharmacy, King Saud University, Riyadh 11451, Saudi Arabia; b Institut für Pharmazie, Bundesstrasse 45 20146, Hamburg, Germany; c Department of Chemistry, University of Malaya, 50603 Kuala Lumpur, Malaysia; d Chemistry Department, Faculty of Science, King Abdulaziz University, PO Box 80203 Jeddah, Saudi Arabia

## Abstract

In the cation of the title salt, C_11_H_12_IN_4_^+^·Cl^−^, the two aromatic rings are oriented to each other at 9.3 (2)°. In the crystal, the two independent Cl^−^ anions lie on twofold rotation axes. N—H⋯Cl hydrogen bonds between the cations and anions generate a supra­molecular layer parallel to (010).

## Related literature

For the synthesis of 6-amino-4-[(4-chloro­phen­yl)amino]-2-methyl­pyridimidine hydro­chloride, see: Craveri & Zoni (1958 ▶). For the synthesis of the reacta­nts, see: Dox (1941 ▶); Foldi *et al.* (1942 ▶).
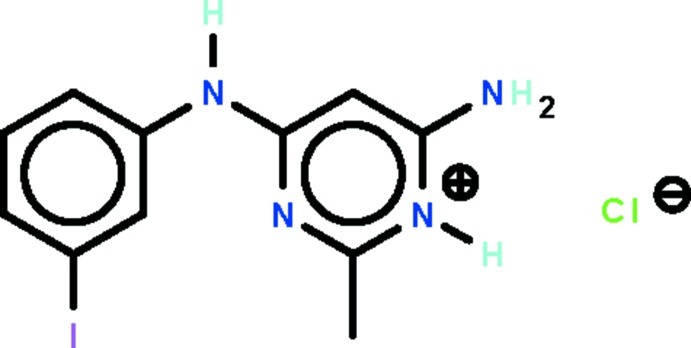


## Experimental

### 

#### Crystal data

C_11_H_12_IN_4_^+^·Cl^−^
*M* *_r_* = 362.60
Orthorhombic, 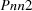

*a* = 12.6323 (5) Å
*b* = 19.8608 (7) Å
*c* = 5.1267 (2) Å
*V* = 1286.23 (8) Å^3^
*Z* = 4
Mo *K*α radiation
μ = 2.68 mm^−1^
*T* = 100 K
0.25 × 0.05 × 0.03 mm

#### Data collection

Agilent SuperNova Dual diffractometer with an Atlas detector
Absorption correction: multi-scan (*CrysAlis PRO*; Agilent, 2012 ▶) *T*_min_ = 0.554, *T*_max_ = 0.924
10009 measured reflections
2981 independent reflections
2833 reflections with *I* > 2σ(*I*)
*R*_int_ = 0.036

#### Refinement

*R*[*F* ^2^ > 2σ(*F* ^2^)] = 0.032
*wR*(*F* ^2^) = 0.078
*S* = 1.07
2981 reflections
172 parameters
5 restraints
H atoms treated by a mixture of independent and constrained refinement
Δρ_max_ = 0.51 e Å^−3^
Δρ_min_ = −0.70 e Å^−3^
Absolute structure: Flack (1983 ▶), 1321 Friedel pairs
Flack parameter: −0.04 (3)

### 

Data collection: *CrysAlis PRO* (Agilent, 2012 ▶); cell refinement: *CrysAlis PRO*; data reduction: *CrysAlis PRO*; program(s) used to solve structure: *SHELXS97* (Sheldrick, 2008 ▶); program(s) used to refine structure: *SHELXL97* (Sheldrick, 2008 ▶); molecular graphics: *X-SEED* (Barbour, 2001 ▶); software used to prepare material for publication: *publCIF* (Westrip, 2010 ▶).

## Supplementary Material

Crystal structure: contains datablock(s) global, I. DOI: 10.1107/S1600536812028401/xu5569sup1.cif

Structure factors: contains datablock(s) I. DOI: 10.1107/S1600536812028401/xu5569Isup2.hkl

Supplementary material file. DOI: 10.1107/S1600536812028401/xu5569Isup3.cml

Additional supplementary materials:  crystallographic information; 3D view; checkCIF report

## Figures and Tables

**Table 1:** Hydrogen-bond geometry (Å, °)

*D*—H⋯*A*	*D*—H	H⋯*A*	*D*⋯*A*	*D*—H⋯*A*
N1—H1⋯Cl1	0.88 (4)	2.48 (4)	3.325 (4)	162 (4)
N3—H3⋯Cl2	0.88 (5)	2.23 (4)	3.096 (4)	168 (4)
N4—H4*A*⋯Cl2	0.89 (4)	2.66 (4)	3.462 (4)	150 (3)
N4—H4*B*⋯Cl1^i^	0.88 (2)	2.64 (3)	3.404 (4)	146 (4)

Symmetry code: (i) 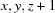.

**Fig. 1.**
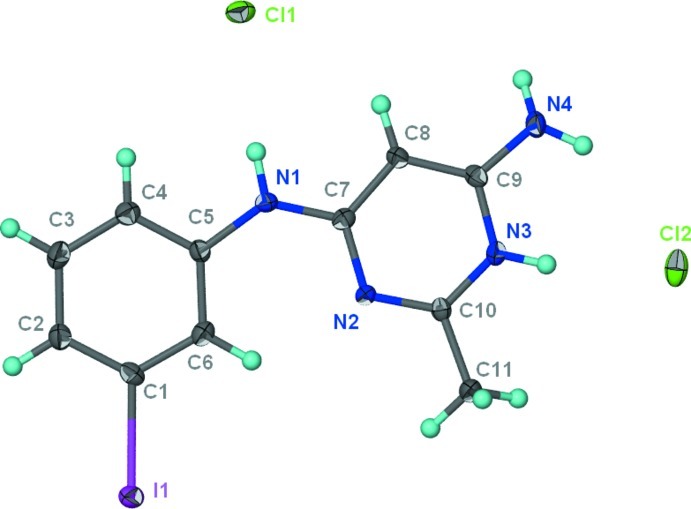
Thermal ellipsoid plot (Barbour, 2001) of C11H12N4I+ Cl- at the 70% probability level; hydrogen atoms are drawn as spheres of arbitrary radius.

## supplementary crystallographic information

### Comment

The synthesis of 6-amino-4-[(3-iodophenyl)amino]-2-methylpyridimidine, which was synthesized with the intention of labeling it with ^99^*^m^*Tc for a study of its biotransformation, requires a small amount of hydrochloric acid as catalyst. A mole of hydrochloric acid is incorporated into the final product, so that the compound is formally 6-amino-4-[(3-iodophenyl)amino]-2-methylpyrimidin-1-ium chloride (Scheme I).

Protonation occurs on the aromatic nitrogen atom that is *para* to the secondary amino substituent. The non-hydrogen atoms of the cation lie on an approximate plane (r.m.s. deviation 0.132 Å); the two aromatic rings were twisted by 9.3 (2) °. The secondary amino and the tertiary pyrimidinium N atoms each forms a hydrogen bonds to a chloride ion to generate a layer parallel to (0 1 0) (Table 1).

### Experimental

A mixture of 4-amino-2-methyl-6-chloropyrimidine (1.55 g, 0.01 mol) and 3-iodoaniline (2.19, 0.01 mol) in absolute ethanol (10 ml) and drop of hydrochloric acid was refluxed for 12 h; 4-amino-2-methyl-6-chloropyrimidine was synthesized from two other reactants (Dox, 1941; Foldi *et al.*, 1942). The reaction mixture was cooled and poured onto ice water. The formed precipitate was filtered, washed with water and recrystallized from ethanol to give the title compound in 60% yield; m.p. 535–537 K. The synthesis duplicates that used for of 6-amino-4-[(4-chlorophenyl)amino]-2-methylpyridimidine, which also exists as a hydrochloride (Craveri & Zoni, 1958).

### Refinement

Carbon-bound H-atoms were placed in calculated positions [C–H 0.95 to 0.98 Å, *U*_iso_(H) 1.2 to 1.5*U*_eq_(C)] and were included in the refinement in the riding model approximation.

The amino H-atoms were located in a difference Fourier map, and were refined with a distance restraint of N–H 0.88±0.01 Å; their temperature factors were refined.

### Crystal data

**Table:**

C_11_H_12_IN_4_^+^·Cl^−^	*F*(000) = 704
*M**_r_* = 362.60	*D*_x_ = 1.872 Mg m^−^^3^
Orthorhombic, *P**n**n*2	Mo *K*α radiation, λ = 0.71073 Å
Hall symbol: P 2 -2n	Cell parameters from 5603 reflections
*a* = 12.6323 (5) Å	θ = 2.6–27.5°
*b* = 19.8608 (7) Å	µ = 2.68 mm^−^^1^
*c* = 5.1267 (2) Å	*T* = 100 K
*V* = 1286.23 (8) Å^3^	Prism, colorless
*Z* = 4	0.25 × 0.05 × 0.03 mm

### Data collection

**Table:**

Agilent SuperNova Dual diffractometer with an Atlas detector	2981 independent reflections
Radiation source: SuperNova (Mo) X-ray Source	2833 reflections with *I* > 2σ(*I*)
Mirror monochromator	*R*_int_ = 0.036
Detector resolution: 10.4041 pixels mm^-1^	θ_max_ = 27.6°, θ_min_ = 2.6°
ω scan	*h* = −16→11
Absorption correction: multi-scan (*CrysAlis PRO*; Agilent, 2012)	*k* = −25→25
*T*_min_ = 0.554, *T*_max_ = 0.924	*l* = −6→6
10009 measured reflections	

### Refinement

**Table:**

Refinement on *F*^2^	Secondary atom site location: difference Fourier map
Least-squares matrix: full	Hydrogen site location: inferred from neighbouring sites
*R*[*F*^2^ > 2σ(*F*^2^)] = 0.032	H atoms treated by a mixture of independent and constrained refinement
*wR*(*F*^2^) = 0.078	*w* = 1/[σ^2^(*F*_o_^2^) + (0.0455*P*)^2^ + 0.098*P*] where *P* = (*F*_o_^2^ + 2*F*_c_^2^)/3
*S* = 1.07	(Δ/σ)_max_ = 0.001
2981 reflections	Δρ_max_ = 0.51 e Å^−^^3^
172 parameters	Δρ_min_ = −0.70 e Å^−^^3^
5 restraints	Absolute structure: Flack (1983), 1321 Friedel pairs
Primary atom site location: structure-invariant direct methods	Flack parameter: −0.04 (3)

### Fractional atomic coordinates and isotropic or equivalent isotropic displacement parameters (Å^2^)

**Table:**

	*x*	*y*	*z*	*U*_iso_*/*U*_eq_	
I1	0.859538 (17)	0.859281 (10)	0.49964 (10)	0.01803 (9)	
Cl1	0.5000	0.5000	0.7100 (3)	0.0165 (3)	
Cl2	1.0000	0.5000	1.8561 (3)	0.0189 (3)	
N1	0.6726 (3)	0.62226 (17)	0.8346 (7)	0.0133 (7)	
H1	0.616 (3)	0.597 (2)	0.815 (12)	0.030 (15)*	
N2	0.8301 (3)	0.63625 (14)	1.0653 (6)	0.0115 (8)	
N3	0.8681 (3)	0.56332 (17)	1.4102 (7)	0.0127 (7)	
H3	0.911 (4)	0.551 (2)	1.537 (8)	0.032 (14)*	
N4	0.7588 (3)	0.47680 (17)	1.5483 (7)	0.0158 (8)	
H4A	0.809 (3)	0.471 (2)	1.667 (7)	0.019 (12)*	
H4B	0.6921 (14)	0.465 (2)	1.559 (12)	0.037 (16)*	
C1	0.7485 (3)	0.78043 (16)	0.4921 (11)	0.0132 (7)	
C2	0.6725 (3)	0.7825 (2)	0.2979 (8)	0.0153 (8)	
H2	0.6724	0.8175	0.1716	0.018*	
C3	0.5960 (3)	0.7318 (2)	0.2927 (8)	0.0174 (9)	
H3A	0.5418	0.7328	0.1644	0.021*	
C4	0.5988 (3)	0.68030 (16)	0.4730 (9)	0.0138 (8)	
H4	0.5464	0.6460	0.4668	0.017*	
C5	0.6770 (3)	0.67774 (17)	0.6634 (8)	0.0116 (7)	
C6	0.7534 (3)	0.72929 (18)	0.6783 (9)	0.0141 (8)	
H6	0.8062	0.7292	0.8101	0.017*	
C7	0.7378 (3)	0.60225 (16)	1.0287 (9)	0.0116 (8)	
C8	0.7081 (3)	0.54705 (17)	1.1824 (8)	0.0126 (7)	
H8	0.6438	0.5235	1.1515	0.015*	
C9	0.7757 (3)	0.52821 (18)	1.3798 (8)	0.0121 (8)	
C10	0.8926 (3)	0.61518 (19)	1.2527 (8)	0.0110 (7)	
C11	0.9960 (4)	0.64830 (19)	1.3029 (9)	0.0190 (9)	
H11A	0.9953	0.6938	1.2292	0.029*	
H11B	1.0528	0.6221	1.2212	0.029*	
H11C	1.0083	0.6509	1.4914	0.029*	

### Atomic displacement parameters (Å^2^)

**Table:**

	*U*^11^	*U*^22^	*U*^33^	*U*^12^	*U*^13^	*U*^23^
I1	0.01513 (14)	0.01341 (13)	0.02556 (15)	−0.00259 (7)	−0.00545 (16)	0.00610 (14)
Cl1	0.0112 (6)	0.0149 (6)	0.0234 (8)	−0.0017 (5)	0.000	0.000
Cl2	0.0187 (7)	0.0262 (7)	0.0117 (7)	0.0092 (5)	0.000	0.000
N1	0.0139 (17)	0.0138 (14)	0.0122 (16)	−0.0050 (14)	−0.0026 (14)	0.0015 (14)
N2	0.0101 (16)	0.0122 (14)	0.012 (2)	−0.0018 (11)	−0.0021 (13)	0.0017 (11)
N3	0.0130 (18)	0.0130 (15)	0.0121 (16)	0.0006 (12)	−0.0030 (13)	0.0017 (13)
N4	0.0135 (16)	0.0214 (15)	0.012 (2)	−0.0011 (12)	−0.0009 (14)	0.0067 (14)
C1	0.0133 (16)	0.0117 (14)	0.0146 (16)	0.0003 (12)	0.003 (2)	0.002 (2)
C2	0.014 (2)	0.0166 (18)	0.015 (2)	0.0006 (16)	−0.0014 (17)	0.0031 (16)
C3	0.016 (2)	0.0180 (18)	0.018 (2)	0.0008 (16)	−0.0055 (17)	0.0016 (17)
C4	0.0132 (16)	0.0123 (14)	0.016 (2)	0.0005 (12)	0.000 (2)	−0.0008 (18)
C5	0.0115 (18)	0.0116 (16)	0.0118 (18)	0.0008 (14)	0.0002 (16)	0.0019 (16)
C6	0.0126 (19)	0.0157 (17)	0.0139 (17)	0.0002 (14)	−0.0026 (16)	−0.0006 (16)
C7	0.0101 (16)	0.0120 (14)	0.013 (2)	0.0001 (11)	−0.0003 (17)	−0.0014 (17)
C8	0.0117 (19)	0.0122 (16)	0.0139 (18)	−0.0014 (14)	−0.0001 (16)	0.0026 (16)
C9	0.016 (2)	0.0103 (15)	0.0099 (18)	−0.0011 (14)	0.0015 (15)	−0.0004 (14)
C10	0.0123 (19)	0.0121 (18)	0.0085 (17)	0.0006 (15)	0.0004 (15)	0.0006 (15)
C11	0.016 (2)	0.022 (2)	0.020 (2)	−0.0062 (16)	−0.0060 (18)	0.0083 (17)

### Geometric parameters (Å, º)

**Table:**

I1—C1	2.103 (3)	C2—H2	0.9500
N1—C7	1.351 (5)	C3—C4	1.380 (6)
N1—C5	1.410 (5)	C3—H3A	0.9500
N1—H1	0.88 (1)	C4—C5	1.390 (6)
N2—C10	1.313 (5)	C4—H4	0.9500
N2—C7	1.360 (5)	C5—C6	1.409 (5)
N3—C10	1.345 (5)	C6—H6	0.9500
N3—C9	1.369 (5)	C7—C8	1.401 (5)
N3—H3	0.88 (1)	C8—C9	1.375 (5)
N4—C9	1.354 (5)	C8—H8	0.9500
N4—H4A	0.88 (1)	C10—C11	1.485 (6)
N4—H4B	0.88 (1)	C11—H11A	0.9800
C1—C2	1.384 (6)	C11—H11B	0.9800
C1—C6	1.395 (6)	C11—H11C	0.9800
C2—C3	1.396 (6)		
			
C7—N1—C5	131.6 (3)	C4—C5—N1	116.0 (3)
C7—N1—H1	114 (4)	C6—C5—N1	124.1 (4)
C5—N1—H1	114 (4)	C1—C6—C5	117.5 (4)
C10—N2—C7	117.3 (3)	C1—C6—H6	121.2
C10—N3—C9	121.2 (3)	C5—C6—H6	121.2
C10—N3—H3	122 (3)	N1—C7—N2	118.5 (3)
C9—N3—H3	117 (3)	N1—C7—C8	118.8 (3)
C9—N4—H4A	115 (3)	N2—C7—C8	122.7 (4)
C9—N4—H4B	113 (4)	C9—C8—C7	117.4 (4)
H4A—N4—H4B	128 (5)	C9—C8—H8	121.3
C2—C1—C6	123.0 (3)	C7—C8—H8	121.3
C2—C1—I1	117.0 (3)	N4—C9—N3	116.5 (3)
C6—C1—I1	120.0 (3)	N4—C9—C8	125.2 (4)
C1—C2—C3	118.2 (4)	N3—C9—C8	118.3 (4)
C1—C2—H2	120.9	N2—C10—N3	123.0 (4)
C3—C2—H2	120.9	N2—C10—C11	121.0 (4)
C4—C3—C2	120.3 (4)	N3—C10—C11	116.0 (4)
C4—C3—H3A	119.8	C10—C11—H11A	109.5
C2—C3—H3A	119.8	C10—C11—H11B	109.5
C3—C4—C5	121.1 (3)	H11A—C11—H11B	109.5
C3—C4—H4	119.5	C10—C11—H11C	109.5
C5—C4—H4	119.5	H11A—C11—H11C	109.5
C4—C5—C6	119.9 (4)	H11B—C11—H11C	109.5
			
C6—C1—C2—C3	1.5 (7)	C5—N1—C7—C8	174.7 (4)
I1—C1—C2—C3	−178.2 (3)	C10—N2—C7—N1	−179.0 (4)
C1—C2—C3—C4	−1.8 (6)	C10—N2—C7—C8	0.4 (6)
C2—C3—C4—C5	0.3 (7)	N1—C7—C8—C9	−179.5 (4)
C3—C4—C5—C6	1.6 (6)	N2—C7—C8—C9	1.1 (6)
C3—C4—C5—N1	−179.2 (4)	C10—N3—C9—N4	179.8 (4)
C7—N1—C5—C4	175.8 (4)	C10—N3—C9—C8	0.3 (6)
C7—N1—C5—C6	−5.0 (7)	C7—C8—C9—N4	179.1 (4)
C2—C1—C6—C5	0.4 (7)	C7—C8—C9—N3	−1.4 (6)
I1—C1—C6—C5	−179.9 (3)	C7—N2—C10—N3	−1.6 (6)
C4—C5—C6—C1	−1.9 (6)	C7—N2—C10—C11	178.6 (4)
N1—C5—C6—C1	179.0 (4)	C9—N3—C10—N2	1.3 (6)
C5—N1—C7—N2	−5.8 (6)	C9—N3—C10—C11	−178.9 (4)

### Hydrogen-bond geometry (Å, º)

**Table:**

*D*—H···*A*	*D*—H	H···*A*	*D*···*A*	*D*—H···*A*
N1—H1···Cl1	0.88 (4)	2.48 (4)	3.325 (4)	162 (4)
N3—H3···Cl2	0.88 (5)	2.23 (4)	3.096 (4)	168 (4)
N4—H4*A*···Cl2	0.89 (4)	2.66 (4)	3.462 (4)	150 (3)
N4—H4*B*···Cl1^i^	0.88 (2)	2.64 (3)	3.404 (4)	146 (4)

Symmetry code: (i) *x*, *y*, *z*+1.
